# A Trans-Hemispheric Migratory Songbird Does Not Advance Spring Schedules or Increase Migration Rate in Response to Record-Setting Temperatures at Breeding Sites

**DOI:** 10.1371/journal.pone.0064587

**Published:** 2013-05-31

**Authors:** Kevin C. Fraser, Cassandra Silverio, Patrick Kramer, Nanette Mickle, Robert Aeppli, Bridget J. M. Stutchbury

**Affiliations:** 1 Department of Biology, York University, Toronto, Ontario, Canada; 2 Woodbridge, Virginia, United States of America; 3 Purple Martin Conservation Association, Erie, Pennsylvania, United States of America; Utrecht University, The Netherlands

## Abstract

The decline of long distance migratory songbirds has been linked to an increasing mismatch between spring arrival date and timing of food availability caused by climate change. It is unclear to what extent individuals can adjust migration timing or en route rate in response to annual variation in temperature at breeding sites. We tracked the ca. 7300 km spring migration of 52 purple martins *Progne subis* from the Amazon basin to two breeding sites in eastern North America. Spring 2012 was the warmest on record in eastern North America, but contrary to predictions, this did not result in earlier departure, faster migration, or earlier arrival at breeding areas compared with earlier years. Temperatures and rainfall in the Amazon basin at the time of departure were not higher in 2012, and conditions along migration routes did not give consistent signals of a warmer spring at the breeding site. Once in North America, individuals likely had limited opportunity to speed up their migration because this final portion of the journey was already very rapid (570 km/d; 4–5 d in duration). Migration timing over the entire journey was best predicted by breeding latitude and sex and was not sensitive to ecological cues (temperature and rainfall amount) at departure from South American overwintering sites or en route, in contrast to recent studies of other songbirds. Our results provide the first direct evidence for a mismatch between higher spring temperatures at breeding sites and departure schedules of individual songbirds, and suggest phenotypic responses to short-term climatic warming may be limited for some species. Further direct-tracking data with greater geographic and temporal scope is needed to test for individual plasticity in response to temperature and rainfall along migratory routes for this, and other, species.

## Introduction

Many animals have shown long-term advancement in spring phenology in response to climate change [1]. The failure of some long-distance migratory songbirds to sufficiently shift arrival date at breeding areas in response to warmer spring temperatures can result in severe population declines [2]. The cause of this inability to shift arrival schedules is much debated [3], and may be driven by constraints on departure date from distant tropical ‘wintering’ areas, and/or by varying conditions and environmental constraints along migratory routes [4]. For long-distance migrants it is unclear if suitable temperature cues are available at tropical wintering sites or if individuals are able to make short-term phenotypic adjustments in departure timing or rate in response to changes in temperature [3]. The amount of rainfall, either at tropical overwintering sites or along migratory routes, may also influence migratory timing of songbirds through its effect on food availability [5], [6], [7]. Recently it has become possible to determine start-to-finish migration timing and rate of small songbirds through direct tracking [8], allowing an unprecedented opportunity to assess phenotypic responses to temperature and rainfall all along migratory routes.

We examined migratory schedules of purple martin *Progne subis*, a declining [9], trans-hemispheric migrant that travels between the Amazon basin and breeding colonies in North America. In 2012, eastern North America experienced the warmest spring since record-keeping began in 1895 [10]. Several studies have shown the existence of large-scale climate and behavioural connectivity between temperate breeding and tropical overwintering sites [11], [12], [13]. Migrants in the tropics may receive signals of anomalous weather events at breeding areas, even while thousands of kilometres away [11]. Weather conditions in 2012 allowed a unique opportunity to examine the extent to which migration schedules and rate are flexible from year to year. Our objectives were to 1) determine if, near the time of departure from tropical non-breeding sites and at passage through key points en route, birds received temperature or rainfall cues of advanced spring and record-setting temperatures at breeding sites in North America, 2) if birds had earlier migration timing (departure, en route, arrival) and faster rate in the warmest spring on record, and, 3) determine if rainfall amount, at departure or en route, was a significant predictor of migration timing and rate.

## Materials and Methods

This study was conducted in accordance with the recommendations of the Ornithological Council 'Guidelines to the Use of Wild Birds in Research' and was approved by the York University Animal Care Committee (Animal Care Protocol Number: 2009-2 W (R1)).

### Geolocator Deployment and Analysis of Light Data

Purple martins were fitted with geolocators (British Antarctic Survey, models MK10, MK12, MK14, MK20) during the nesting period (2007–2011, n = 228) at two eastern breeding locations (Pennsylvania, Virginia; Table S1). Geolocators were retrieved (n = 73; 52 with spring migration data) in the year following deployment. Purple martins fitted with geolocators did not have a lower return rate than those not carrying geolocators [14]. Raw light data were corrected for clock drift using BASTrak and analyzed using TransEdit (British Antarctic Survey). We manually verified a sharp transition at each sunrise and sunset and deleted obvious shading events during the daytime. We used a light-level threshold of 32 (MK14, MK10) or 5 (MK12, MK20) to define sunrise and sunset transitions, and used live calibration data from birds after nesting but prior to migration to determine the average sun elevation that corresponded with this light threshold level at the breeding site. Sun elevation values were averaged across individuals within each year to better represent average conditions for migrating birds at unknown locations. Latitude was not determined for 15 days before and after the spring equinox when day length is similar everywhere. During this period, positions were estimated using longitude [8]. This is appropriate for spring migration in this species, as migratory routes have a large longitudinal component [15]. Latitude and longitude coordinates were calculated with Locator software (British Antarctic Survey) using midnight locations because purple martins are primarily diurnal migrants. Movements away from tropical roosts consistent with spring migration, and from one stopover location to another (>200 km latitude, >100 km longitude), were defined as migration. Arrival at breeding sites was considered to have occurred when the latitude and longitude ceased to shift in a direction consistent with spring migration and fluctuated around a narrow range of values (less than 2 degrees longitude), consistent with a stationary bird, and frequent shading events indicated use of nesting boxes. To estimate geolocator accuracy we calculated location for two weeks after nesting but prior to migration and compared that with the known roost or breeding colony location. Average geolocator accuracy at breeding sites prior to migration, ranged from 20–60 km for latitude, and 20–75 km for longitude [14].

### Temperature Data

We compared average maximum daily temperatures at two breeding areas in 2012 versus prior years (2008–2011 PA; 2011 VA) during the main departure period from Brazil (March 15 to April 15). We also compared spring warmth sum (sum of maximum daily temperatures [16]) over the same period to provide a cumulative estimate of differences between years. The average daily maximum temperature 10 days before and after the median passage date [4] of each population in 2012 was compared using t-tests to prior years at the core wintering area (Manaus Brazil), and at three points en route: 1) Panama (Panama City); 2) Yucatan Peninsula (Merida, Mexico), 94% of birds crossed the Yucatan Peninsula; and 3) Along the U.S. Gulf coast (PA population: Mobile, Alabama; VA: Panama City, Florida). Total rainfall amount was determined for the month (30 d) prior to median migration passage date at each of the 4 locations above because rainfall in the month leading up to migration is expected to have the greatest influence on migration timing via effects on food supply [5]. Temperature and rainfall data are from the National Oceanic and Atmospheric Administration [17].

### Statistical Tests of Migration Timing and Rate

First, we used univariate tests to compare migration timing (t-test) and rate (ANOVA) between 2012 and earlier years, for each breeding population. Next, we used linear mixed-effects models fit by REML to assess the influence of temperature and rainfall on migration timing and rate at the core wintering and stopover locations. We examined timing at four locations (Brazil, Panama, Yucatan, and breeding site) and rate in three migration zones (South, Central and North America). We fit a separate timing and rate model for each location and zone. For arrival date at stopover sites and migration rate in each zone, we used temperature and rainfall amounts at the preceding site as factors, reasoning that timing and rate would be most influenced by conditions prior to arrival (i.e. at the previous site). The full models included fixed factors of population (PA or VA), sex, rainfall and temperature with a random effect of year. We dropped individual explanatory variables one by one, then used Akaike’s Information Criterion weights to select the best-fit model. To assess the significance of each variable, we removed them one by one from the full model, then compared each reduced model to the full model using a likelihood ratio test. All analyses were conducted using R [18].

## Results

**Figure 1:**
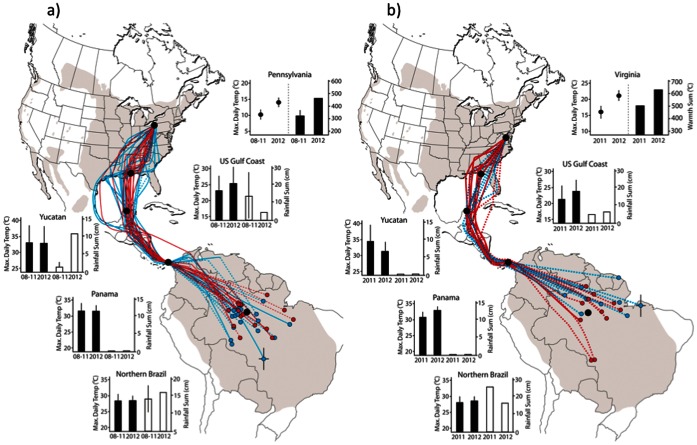
Spring migration routes and en route temperatures and rainfall for purple martins. Birds were tracked from two breeding populations a) Pennsylvania (41.8°N, 79.9°W) and b) Virginia (38.61°N, 77.26°W). Spring routes shown in red (2012) and blue lines (2008-11 PA; 2011 VA). Dashed lines show estimated route based on longitude when latitude was uncertain due to equinox. Black circles show locations of en route temperature and rainfall measurements and correspond to adjacent graphs showing mean ± SD temperature during passage (10 d pre-and post- median passage date) and rainfall sum (30 d pre-median departure/passage date; ± SD where multiple years). Breeding site graphs (Pennsylvania and Virginia) show maximum daily temperature and spring warmth sum at breeding sites March 15– April 15 (circles show mean, bars standard deviation). Last winter roost locations in South America before spring migration are shown by red and blue circles. Error bars, shown for one bird on each map, are typical standard deviation in latitude and longitude for estimated winter locations.

**Figure 2:**
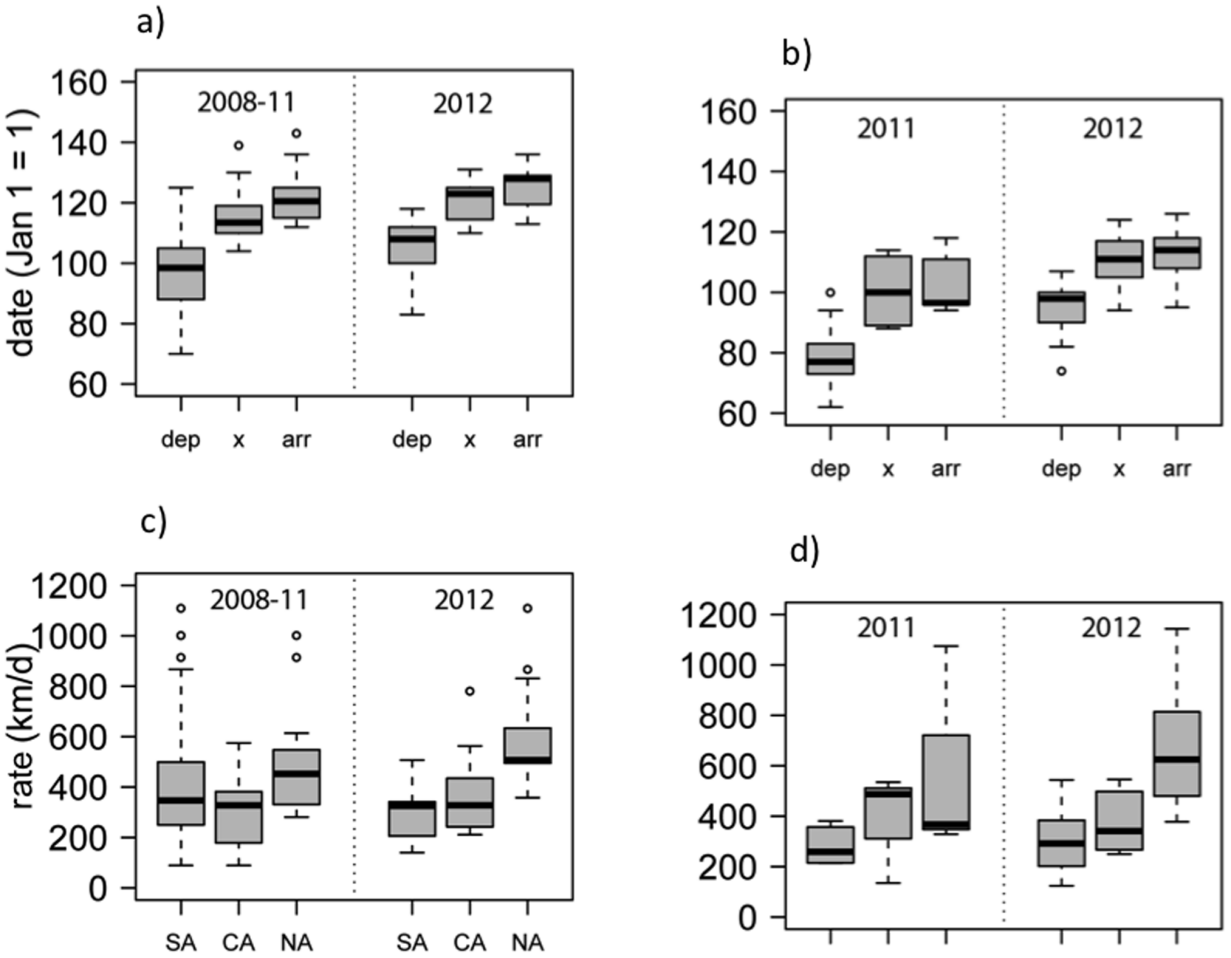
Migration rate of purple martins in record warm year 2012 versus prior year(s). Timing of spring migration at departure (dep), date crossing 23.4°N (x), breeding arrival date (arr) for breeding populations from a) Pennsylvania and b) Virginia. Spring migration rate during three stages en route (South America, SA; Central America, CA; North America, NA), c) Pennsylvania (2008-11, n = 18; 2012, n = 15) and d) Virginia (2011, SA n = 9, CA n = 6, NA n = 8; 2012, n = 10).

In 2012, maximum daily spring temperature was significantly higher at breeding sites for both populations (Fig. 1a–b; PA: *t* = −3.42, *df* = 49.38, *P = *0.001, VA: *t* = −3.09, *df = *54.51, *P = *0.003) and spring warmth sum was 46% (PA) and 25% (VA) higher than previous years. However, in 2012 birds departed significantly later, not earlier, from wintering sites (PA: *t = *2.29, *df = *30.06, *P = *0.03; VA: *t = *2.99, *df = *15.35, *P = *0.008) and there was no difference between years in the timing of crossing the Gulf of Mexico (23.4°N) or arrival at breeding sites (Fig. 2a–b). The Virginia population, as expected, had earlier departure, passage, and arrival dates than the more northern Pennsylvania population.

Rate of spring migration varied significantly (PA: *F_1,96_* = 30.52, *P*<0.0001; VA: *F_1,39_* = 25.61, *P*<0.0001) during passage through South America (PA: 310 km/d ±30; VA: 289 km/d ±28), Central America (PA: 333 km/d ±25; VA: 370 km/d ±37) and North America (PA: 538 km/d ±35; VA: 649 km/d ±75). Migration rate was not significantly faster in 2012 versus prior years (Fig. 2c–d) for any stage of migration (PA: *F_1,96_* = 1.75, *P = *0.19; VA: *F_1,39_* = 0.05, *P = *0.83). Migration duration from the Yucatan Peninsula to the breeding colony was only 4–5 days on average (PA: 5±0.35; VA: 4±0.55).

Temperatures at the core overwintering area [14] in northern Brazil were not significantly warmer in 2012 (Table 1; Fig. 1). Temperatures were generally similar between years along migratory routes, during the peak passage period specific to each population. However, there were warmer temperatures in 2012 at Panama for birds from the Virginia breeding population and a trend for higher temperatures in 2012 for both populations once birds reached the U.S. Gulf Coast (Table 1; Fig. 1). Rainfall at wintering and passage sites was not consistently higher in 2012.

**Table 1:** Temperatures experienced by purple martins during peak passage times along migratory routes.

En route location and breeding population tracked	*pre-2012*	*2012*	*t*	*P*
**U.S. Gulf Coast**				
Pennsylvania	23.1±4.7	25.2±4.9	−1.72	0.097
Virginia	21.8±3.9	24.1±3.6	−1.95	0.059
**Yucatan Peninsula**				
Pennsylvania	33.2±5.3	32.9±5.8	−0.21	0.84
Virginia	34.3±5.4	31.0±3.2	2.34	0.03
**Panama City**				
Pennsylvania	31.8±1.9	32.0±1.9	−0.45	0.65
Virginia	31.0±1.9	32.7±1.0	−3.59	0.001
**Manaus, Brazil**				
Pennsylvania	28.0±2.1	28.1±1.9	−0.03	0.98
Virginia	27.9±2.0	28.2±1.8	−0.50	0.62

Mean (± SD) maximum daily temperature 10 days before and after median departure date from Brazil, and at passage through Panama, the Yucatan Peninsula, and the U.S. Gulf Coast for birds tracked from two breeding populations (Pennsylvania, n = 33; Virginia, n = 19). Statistics reported from t-tests.

Based on our model results, neither rainfall amount or temperature were significant factors predicting departure date from South American overwintering sites in the Amazon Basin. Our best-fit models of migration departure date included only sex and breeding location (PA or VA) and both were significant in the model (sex: r^2^ = 4.71, df = 1; breeding location: r^2^ = 16.27, df = 1, P<0.0001). On average, males left 5 days earlier than females and individuals breeding at the more southerly Virginia colony departed 14 days earlier than birds breeding in Pennsylvania. We had similar results for migration timing en route at Panama, Yucatan and breeding arrival where best-models included only sex and breeding location. Both factors were significant in the models except for sex at the Yucatan (r^2^ = 3.77, df = 1, P<0.052). None of the factors in our models of migration rate during each of the three stages of migration (SA, CA, NA) were significant, illustrating that birds did not differ their rate in response to temperature or rainfall en route and that rate did not differ by breeding location or sex.

## Discussion

We found no evidence that purple martins advanced their departure date from South America, or increased their migration rate, during the warmest spring on record at breeding sites in eastern North America. Consistent temperature and rainfall cues were not present in South or Central America, and these weather variables were not significant predictors of departure date and en route migration timing and rate. These results, based on direct-tracking of purple martin, are surprising and contrast recent papers that show that some species may be able to increase rate in response to conditions en route [19], [20], [21] or in general enact a strong phenotypic response to changing weather and environmental conditions [5], [6], [7], [22], [23].

Spring departure date from tropical overwintering sites of a forest songbird was highly repeatable between years, suggesting limited phenotypic plasticity in some species [24]. Inflexible departure schedules in warm springs could be a result of mostly endogenous control of migration timing [25], [26], [27] but may also reflect limited environmental cues available to long-distance migrants about conditions at the breeding grounds [11], [12], [13]. Purple martins apparently received limited or conflicting environmental cues of an early spring at breeding sites while still at wintering sites and en route. Once en route, birds received no (PA population) or inconsistent (VA population) temperature cues of the warm spring until they reach the U.S. Gulf coast. However, most martins travelled more than 400 km/d during their final 4–5 day spring passage to their breeding sites, supporting the hypothesis that long-distance migrants have little opportunity to advance their rate of migration in response to temperature cues at the final stage of their journey [4]. Our model results suggest that migration timing and rate in purple martin is not highly sensitive to short-term variation in temperature and rainfall. We suggest that multiple years of increasing spring temperatures may be required to generate an adaptive response of earlier breeding arrival timing of purple martins, via selection for earlier departing individuals. Further direct-tracking data with greater geographic and temporal scope, and repeat tracking of individual birds, is needed to better understand individual plasticity in response to temperature and rainfall along migratory routes.

Aerial insectivores, particularly species migrating longer distances and populations breeding in the north, are experiencing strong population declines [9]. We suggest that this could be driven by constraints on spring departure date, the absence of strong and consistent temperature cues, and low opportunity for rate adjustments during migration leading to a mismatch between arrival date and optimal breeding conditions. Recent direct tracking of other species has shown a strong correlation between departure and arrival date as well as rapid spring migration [28], suggesting many long-distance migrants may have limited opportunities to respond to short-term climatic warming.

## Supporting Information

Table S1 **Geolocator deployment locations, year, type, number of units deployed, geolocators retrieved (does not include birds who lost tags) and total sample size for spring migration (excludes tags that failed prior to spring migration).** Most (75%) geolocators were 1.1 g with a <10 mm stalk (MK10S, British Antarctic Survey) and were mounted using a leg-loop backpack harness [1], [2].
(DOCX)
