# Supplementary material for: A Trans-Hemispheric Migratory Songbird Does Not Advance Spring Schedules or Increase Migration Rate in Response to Record-Setting Temperatures at Breeding Sites
Source: PLoS One. 2013 May 31;8(5):e64587. doi: 10.1371/journal.pone.0064587 (PMC3669305; doi:10.1371/journal.pone.0064587)
Supplement: Table S1 — Geolocator deployment locations, year, type, number of units deployed, geolocators retrieved (does not include birds who lost tags) and total sample size for spring migration (excludes tags that failed prior to spring migration). Most (75%) geolocators were 1.1 g with a <10 mm stalk (MK10S, British Antarctic Survey) and were mounted using a leg-loop backpack harness [1], [2]. (DOCX) [file pone.0064587.s001.docx]

**Supporting Table S1. Geolocator deployments.**

| **Year(s)**  **deployed** | **Latitude** | **Longitude** | **Weight (g), stalk length (mm), model** | **Number Deployed** | **Geolocators Retrieved** | **Sample size spring migration** |
| --- | --- | --- | --- | --- | --- | --- |
| **Pennsylvania** | | | | | | |
| 2007-8 | 41° 53’ 08”N | 80°07’ 46”W | 1.1-1.5, 20, MK 10S/14S | 40 | 5 | 5 |
| 2009-10 | 41° 53’ 08”N  42° 08’ 59”N | 80°07’ 46”W  80°07’ 58”W | 0.6-1.1, 0-8, MK 20/10S | 47 | 18 | 13 |
| 2011 | 42° 08’ 59”N | 80°07’ 58”W | 1.2, 9, MK10S | 54 | 19 | 15 |
| **Virginia** | | | | | | |
| 2010 | 38° 36’47” N | 77°15’46” W | 0.9-1.1, 0-8, MK12 | 37 | 19 | 9 |
| 2011 | 38° 36’47” N | 77°15’46” W | 1.2, 9, MK10S | 50 | 12 | 10 |
| **TOTAL** |  |  |  | **228** | **73** | **52** |

**Literature Cited**

1. Stutchbury BJM, Tarof SA, Done T, Gow E, Kramer PM, et al. (2009) Tracking long-distance songbird migration by using geolocators. Science 323: 896-896.

2. Rappole JH, Tipton AR (1991) New harness design for attachment of radio transmitters to small passerines. Journal of Field Ornithology 62: 335-337.
